# Survey on the current status of photoaging in young Asian women and intervention effects of non-ablative bipolar radiofrequency combined with ablative fractional CO₂ laser: a retrospective study

**DOI:** 10.3389/fmed.2026.1684986

**Published:** 2026-05-01

**Authors:** Xie Qiu, Wei Zhang, Xiaofeng Duan

**Affiliations:** 1 Department of Dermatology, Ningbo Medical Center Lihuili Hospital, Ningbo, Zhejiang Province, China; 2 Department of Cosmetic Dermatology, Changshu Shangxi Medical Aesthetics Clinic, Suzhou, Jiangsu Province, China; 3 Department of College of Aesthetic Medicine, Yichun University, Yichun, Jiangxi, China

**Keywords:** ablative fractional CO_2_ laser, combination therapy, Fitzpatrick skin type, non-ablative bipolar radiofrequency, skin photoaging, young Asian women

## Abstract

**Objective:**

This study aimed to investigate the current degree of skin photoaging in young Asian women and to explore the intervention effects of non-ablative bipolar radiofrequency (RF) combined with ablative fractional CO₂ laser.

**Methods:**

A retrospective study was conducted on 202 young Asian female patients (aged 18–35 years) with photodamaged skin who attended our hospital between March 2023 and March 2024. The patients were divided into two groups based on the intervention program: the RF-alone group (*n* = 80, non-ablative bipolar radiofrequency treatment only) and the combined group (*n* = 122, non-ablative bipolar radiofrequency combined with ablative fractional CO₂ laser). The Glogau photoaging classification, Fitzpatrick skin typing, clinical efficacy, symptom scores, global photoaging score (GPS), and adverse reactions were compared between the two groups.

**Results:**

Among the 202 young Asian female patients with photodamaged skin, the Glogau classification showed 144 cases (71.29%) classified as Grade I and 58 cases (28.71%) classified as Grade II. Fitzpatrick skin typing revealed predominantly Types I–II (approximately 71%): Type I 76 cases (37.62%), Type II 68 cases (33.66%), Type III 36 cases (17.82%), and Type IV 22 cases (10.89%). The combined group showed a significantly higher total effective rate than the RF-alone group (95.08% vs. 78.75%; χ^2^ = 12.773, *p* < 0.001; risk difference: 16.33, 95% CI: 7.24–25.42%). Post-treatment symptom scores for pore size, hyperpigmentation, skin texture, and skin color were significantly lower in the combined group (all *p* < 0.05). GPS scores were also significantly lower in the combined group (1.58 ± 0.50 vs. 2.24 ± 0.56; mean difference: −0.66, 95% CI: −0.80 to −0.52, *p* < 0.001). No significant difference was observed in adverse reaction rates between the groups (4.10% vs. 3.75%, *p* > 0.05).

**Conclusion:**

The degree of skin photoaging in young Asian women is predominantly mild to moderate, with greater sensitivity to ultraviolet radiation characteristic of Fitzpatrick Types I–II. Non-ablative bipolar radiofrequency combined with an ablative fractional CO₂ laser demonstrates superior efficacy in improving photoaging symptoms compared with RF alone, without a significantly increase in adverse reactions. This combination approach represents a safe and effective intervention strategy for the management of early photoaging in young Asian women.

## 1 Introduction

Skin aging comprises both intrinsic (chronological) aging and extrinsic aging. Intrinsic aging occurs naturally over time, whereas extrinsic aging results from environmental factors, including sun exposure, cigarette smoking, and air pollution (1). Among these external factors, ultraviolet (UV) radiation represents the primary cause of extrinsic skin aging, commonly termed photoaging. Although ultraviolet, visible, and infrared light all contribute to photoaging, UV radiation exerts the most pronounced effects, making it the predominant factor in clinical photoaging assessment (2). Among young women, inadequate sun protection and adverse lifestyle habits can lead to premature photoaging, manifesting as roughness, dryness, laxity, deepened wrinkles, hyperpigmentation, and, in severe cases, benign or malignant cutaneous neoplasms, thereby affecting facial esthetics and psychological wellbeing (3).

Contemporary esthetic medicine offers various modalities for photoaging treatment. Non-ablative bipolar radiofrequency (RF) generates controlled dermal heating through electromagnetic energy delivery, inducing collagen contraction and neocollagenesis via thermal stimulation of fibroblasts (4, 5). The bipolar configuration provides precise energy deposition within the dermis while minimizing epidermal damage. However, RF monotherapy demonstrates limited efficacy for addressing epidermal pigmentary abnormalities and superficial textural changes, often necessitating adjunctive treatments (6). Ablative fractional CO₂ laser technology creates microscopic thermal zones (MTZs) surrounded by viable tissue reservoirs, enabling rapid re-epithelialization while stimulating collagen remodeling throughout both the epidermis and dermis (7, 8). The fractional delivery pattern minimizes recovery time and adverse events compared to traditional ablative resurfacing. Recent evidence suggests that combining these complementary modalities may provide synergistic benefits: RF addresses deep dermal laxity and stimulates volumetric collagen production, while fractional CO₂ laser targets superficial textural irregularities, dyschromia, and fine rhytides (9, 10). This multi-depth approach theoretically optimizes photoaging correction by simultaneously addressing different pathophysiological targets.

Despite growing clinical utilization, evidence examining the combination of RF and fractional CO₂ laser therapy in young Asian women with early photoaging remains limited. Asian skin, characterized by higher melanin content and distinct structural properties, presents unique considerations regarding treatment parameters and adverse event profiles, particularly the risk of post-inflammatory hyperpigmentation (PIH) (11). This study hypothesizes that non-ablative bipolar RF combined with ablative fractional CO₂ laser provides superior improvement in photoaging compared to RF alone in young Asian women, with an acceptable safety profile. Accordingly, this retrospective analysis aimed to characterize photoaging patterns in young Asian women and evaluate the comparative efficacy and safety of combination therapy versus RF monotherapy.

## 2 Materials and methods

### 2.1 Ethics statement

This study was approved by the Ethics Committee of Ningbo Medical Center at Lihuili Hospital (Approval No. NBLH-2023-EC-078, approved 15 December 2022). All research was conducted in accordance with the Declaration of Helsinki. Due to the retrospective nature of the study using de-identified patient data, informed consent was waived per institutional guidelines, as this posed no medical risk or adverse effects to the patients.

### 2.2 Study design and participants

**Figure 1:**
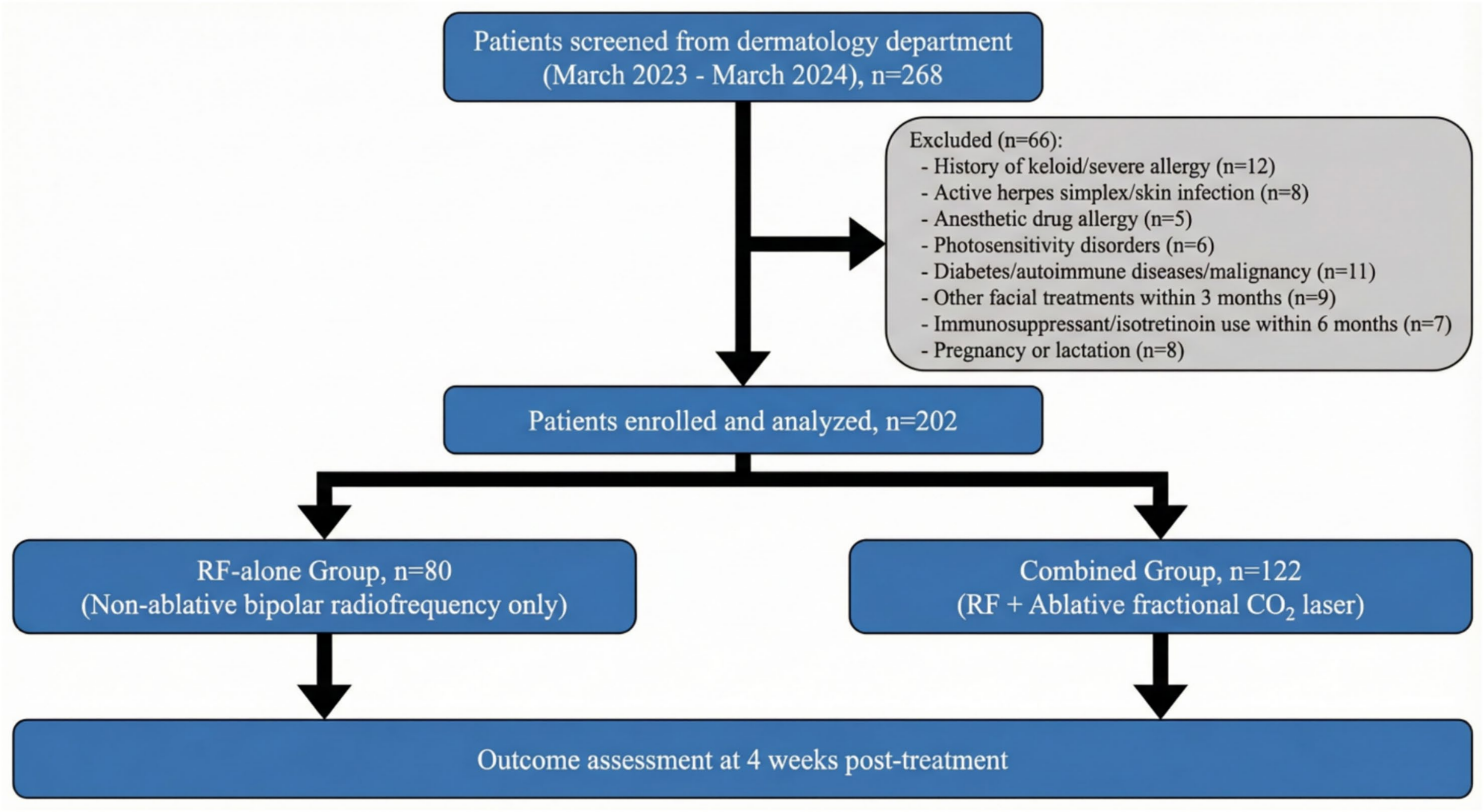
The strengthening the reporting of observational studies in epidemiology (STROBE) flow diagram.

This retrospective analysis included 202 young Asian female patients with photodamaged skin who attended our dermatology department between March 2023 and March 2024. Patient data were extracted from the electronic medical record system. Based on the treatment protocol, patients were categorized into the RF-alone group (*n* = 80, receiving non-ablative bipolar radiofrequency treatment only) and the combined group (*n* = 122, receiving non-ablative bipolar radiofrequency combined with an ablative fractional CO₂ laser). The study flow is presented in Figure 1.

### 2.3 Inclusion and exclusion criteria

The inclusion criteria comprised (1) a clinical diagnosis of photodamaged skin with manifestations including dryness, pigmentation, and enlarged pores, and a Glogau photoaging classification of ≥ Grade I (12); (2) female sex, aged 18–35 years; (3) voluntary request for facial rejuvenation treatment; and (4) adequate compliance and the ability to adhere to treatment protocols. The exclusion criteria included (1) a history of keloid or severe cutaneous allergy; (2) active herpes simplex, active skin infection, or open facial wounds; (3) allergy to anesthetic drugs; (4) photosensitivity disorders; (5) diabetes mellitus, autoimmune diseases, hematological disorders, or malignancy; (6) receipt of other facial cosmetic treatments within 3 months prior to enrollment; (7) use of immunosuppressants, glucocorticoids, isotretinoin, or exfoliating cosmetics within 6 months; (8) a tendency toward keloid scarring; and (9) pregnancy or lactation.

### 2.4 Treatment protocols

**Figure 2:**
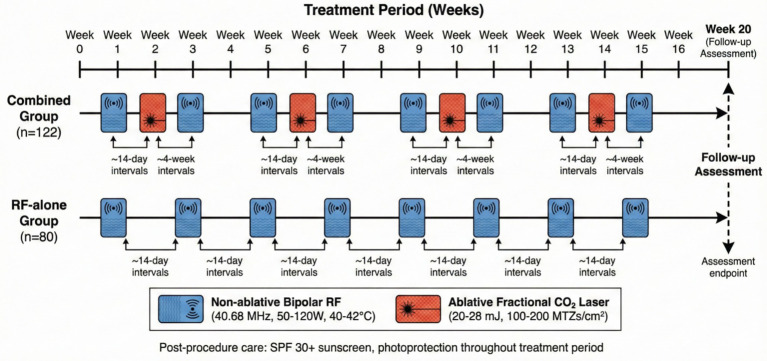
Procedural schematic illustrating the treatment timeline. The diagram shows the sequencing and alternation between non-ablative bipolar radiofrequency (RF) sessions (weeks 1, 3, 5, 7, 9, 11, 13, and 15) and ablative fractional CO₂ laser sessions (weeks 2, 6, 10, and 14) in the combined treatment group. The RF-alone group received only the RF sessions at the same time points.

The treatment timeline and sequencing of interventions are illustrated in Figure 2.

*Non-ablative Bipolar Radiofrequency Protocol:* The Accent Pro Deep Blue Thermoplastic Radiofrequency system (Alma Lasers, Israel) was used in the bipolar, non-ablative, non-fractional mode. The device specifications were as follows: an emission frequency of 40.68 MHz, a power range of 50–120 W (adjusted according to treatment area and skin type), and a target tissue temperature of 40–42 °C, maintained through integrated real-time impedance and temperature monitoring. Treatment was performed using the UniLarge applicator (40 mm × 40 mm contact area) with an aqueous coupling gel. The treatment endpoint was a sustained tissue temperature of 40–42 °C for 3–5 min per treatment zone, as confirmed by integrated thermal feedback. The treatment procedure covered the full face with 2–3 passes per area. Sessions were conducted twice monthly (approximately 14-day intervals) for 4 consecutive months (totaling 8 sessions). Post-procedure care included the immediate application of a hydrating skincare serum.

*Ablative Fractional CO₂ Laser Protocol:* The JLT-100A Carbon Dioxide Laser Therapeutic Instrument (Wuhan JinLaiTe Optoelectronics Co., Ltd., China) was used in the ablative fractional mode. Prior to treatment, a topical 2.5% lidocaine cream (Tongfang Pharmaceutical Co., Ltd.; NMPA approval H20063466) was applied to the treatment area for 60 min. Laser parameters were individualized accordin to the severity of photoaging: for patients with fine lines and mild elastic tissue deformation, energy was set at 20–25 mJ per microbeam, with a frequency of 900 Hz, spot spacing of 0.4–0.6 mm, scanning time of 0.1–0.2 ms, fluence of approximately 8–12 J/cm^2^, spot diameter of 120 μm, and density of 100–150 MTZs/cm^2^, with 1–2 passes and for patients with moderate wrinkles, pigmentary changes, or reduced skin elasticity, energy was set at 22–28 mJ per microbeam, with a frequency of 900 Hz, spot spacing of 0.2–0.6 mm, scanning time of 0.1–0.3 ms, fluence of approximately 10–15 J/cm^2^, and density of 150–200 MTZs/cm^2^, with 1–2 passes. The treatment was delivered in micro-ablative mode. Fractional CO₂ laser was performed once monthly for 4 consecutive months (totaling 4 sessions).

*Combined Treatment Sequencing:* In the combined group, treatments were administered in alternating sessions rather than as same-day sequential application. Specifically, RF treatments were administered on weeks 1, 3, 5, 7, 9, 11, 13, and 15, while fractional CO₂ laser treatments were administered on weeks 2, 6, 10, and 14, allowing adequate recovery between modalities. This alternating schedule was designed to minimize cumulative thermal injury while maximizing collagen remodeling stimulus. Both groups received standardized post-procedure instructions, including refraining from facial washing for 24 h, avoiding cosmetics for 48 h, strict photoprotection (SPF 30 + sunscreen and physical barriers), and avoidance of direct sun exposure throughout the treatment period.

### 2.5 Data collection

General demographic data, including age, place of residence, body mass index, education level, menarche age, and daily sun exposure duration, were extracted from electronic medical records. Baseline disease characteristics, including the Glogau photoaging classification and the Fitzpatrick skin phototype, were recorded. Clinical outcomes were assessed 4 weeks following completion of the treatment protocol.

### 2.6 Photoaging assessment

The Glogau photoaging classification was applied as follows: Grade I (mild photoaging): no obvious wrinkles, slight pigmentation irregularity (e.g., freckles), no actinic keratoses, and minimal makeup use required; Grade II (moderate photoaging): dynamic wrinkles appearing with facial movement (e.g., crow’s feet), early pigmented lesions (e.g., solar lentigines), and moderate makeup use required; Grade III (severe photoaging): static wrinkles persisting at rest (e.g., glabellar lines and nasolabial folds), obvious dyspigmentation (e.g., melasma), skin roughness, telangiectasia, and heavy makeup use required; and Grade IV (very severe photoaging): diffuse wrinkles, skin laxity and ptosis, sallow-yellow complexion, actinic keratoses or precancerous lesions, and makeup insufficient for coverage (12).

### 2.7 Skin phototype assessment

The Fitzpatrick skin phototype classification categorized patients as follows: Type I: always burns, never tans, and constitutive color white; Type II: usually burns, tans minimally, constitutive color white; Type III: sometimes burns, tans uniformly, and constitutive color white; Type IV: rarely burns, tans moderately, and constitutive color light brown; Type V: very rarely burns, tans profusely, and constitutive color brown; and Type VI: never burns, deeply pigmented, and constitutive color dark brown to black (5).

### 2.8 Clinical efficacy evaluation

Clinical efficacy was determined using the skin improvement rate formula: Improvement rate = [(pre-treatment score − post-treatment score)/pre-treatment score] × 100%. The outcomes were categorized as: excellent (improvement rate >75%), good (51–75%), fair (26–50%), or poor (≤25%). The total effective rate was calculated as: Total effective rate = [(number of excellent + good + fair cases)/total cases] × 100% (13).

### 2.9 Symptom scoring

Two experienced dermatologists (each with >10 years of clinical experience) independently evaluated symptom severity before and after treatment using the VISIA Complexion Analysis System (7th generation, Canfield Scientific Inc., United States). Evaluated parameters included pore size, hyperpigmentation, skin texture, and skin color, each scored on a scale of 0–9 (0–3: mild; 4–6: moderate; 7–9: severe). Higher scores indicated greater symptom severity. Inter-rater reliability was assessed using Cohen’s weighted kappa coefficient, yielding *κ* = 0.82 (95% CI: 0.76–0.88), indicating substantial agreement. Discrepancies were resolved by consensus review.

### 2.10 Global photoaging score

The GPS was evaluated by the same two blinded dermatologists based on standardized clinical photographs. The scoring criteria were as follows: 0 points: no fine wrinkles, pigmentation irregularity, or textural roughness in the cheeks, the forehead, or the periorbital areas, and skin palpation was smooth; 1 point: visible roughness, dyspigmentation, or fine wrinkles in one of the three facial zones, or combined findings within one zone; 2 points: visible changes in two of the three zones, or combined findings in one zone; 3 points: visible changes in all three zones, or combined findings in two zones; and 4 points: changes exceeding 3-point criteria (14). Lower GPSs indicated better photoaging improvement. Inter-rater reliability for GPS assessment yielded *κ* = 0.79 (95% CI: 0.72–0.86).

### 2.11 Adverse reaction assessment

Adverse reactions monitored during and after treatment included skin dryness, pruritus, burning sensation, erythema, and post-inflammatory hyperpigmentation. The adverse reaction rate was calculated as: (number of patients with adverse reactions/total number of patients) × 100%.

### 2.12 Statistical analysis

Statistical analyses were performed using SPSS 25.0 (IBM Corp., Armonk, NY, United States). Categorical variables were expressed as counts (percentages) and analyzed using chi-square tests or Fisher’s exact test when expected cell counts were <5. Continuous variables conforming to normal distribution were expressed as mean ± standard deviation (x̄ ± s) and compared using independent samples t-tests. Effect sizes were calculated as mean differences with 95% confidence intervals (CI) for continuous outcomes and risk differences with 95% CI for categorical outcomes. Given the exploratory nature of this study with a single primary outcome (total effective rate), no formal multiple comparison adjustment was applied; however, secondary outcomes should be interpreted with caution. Statistical significance was set at a two-tailed *p* < 0.05. A *post hoc* power analysis indicated that, with the observed effect size for total effective rate (16.33% difference), our sample provided >90% statistical power to detect this difference at *α* = 0.05.

## 3 Results

### 3.1 General characteristics

The 202 young Asian female patients with photoaging were aged 18–35 years, with a mean of 28.40 ± 5.32 years. Regarding residence, 80 patients (39.60%) lived in rural areas, and 122 (60.40%) resided in urban areas. The body mass index ranged from 20.6 to 24.8 kg/m^2^, with a mean of 22.10 ± 1.64 kg/m^2^. Education-level distribution showed 62 patients (30.69%) with junior high school education or below and 140 (69.31%) with high school education or above. Menarche age ranged from 11 to 15 years, with a mean age of 13.36 ± 1.20 years. Daily sun exposure duration ranged from 1 to 8 h, with a mean duration of 3.98 ± 0.56 h.

### 3.2 Distribution of the Glogau photoaging classification and the Fitzpatrick skin phototype

**Figure 3:**
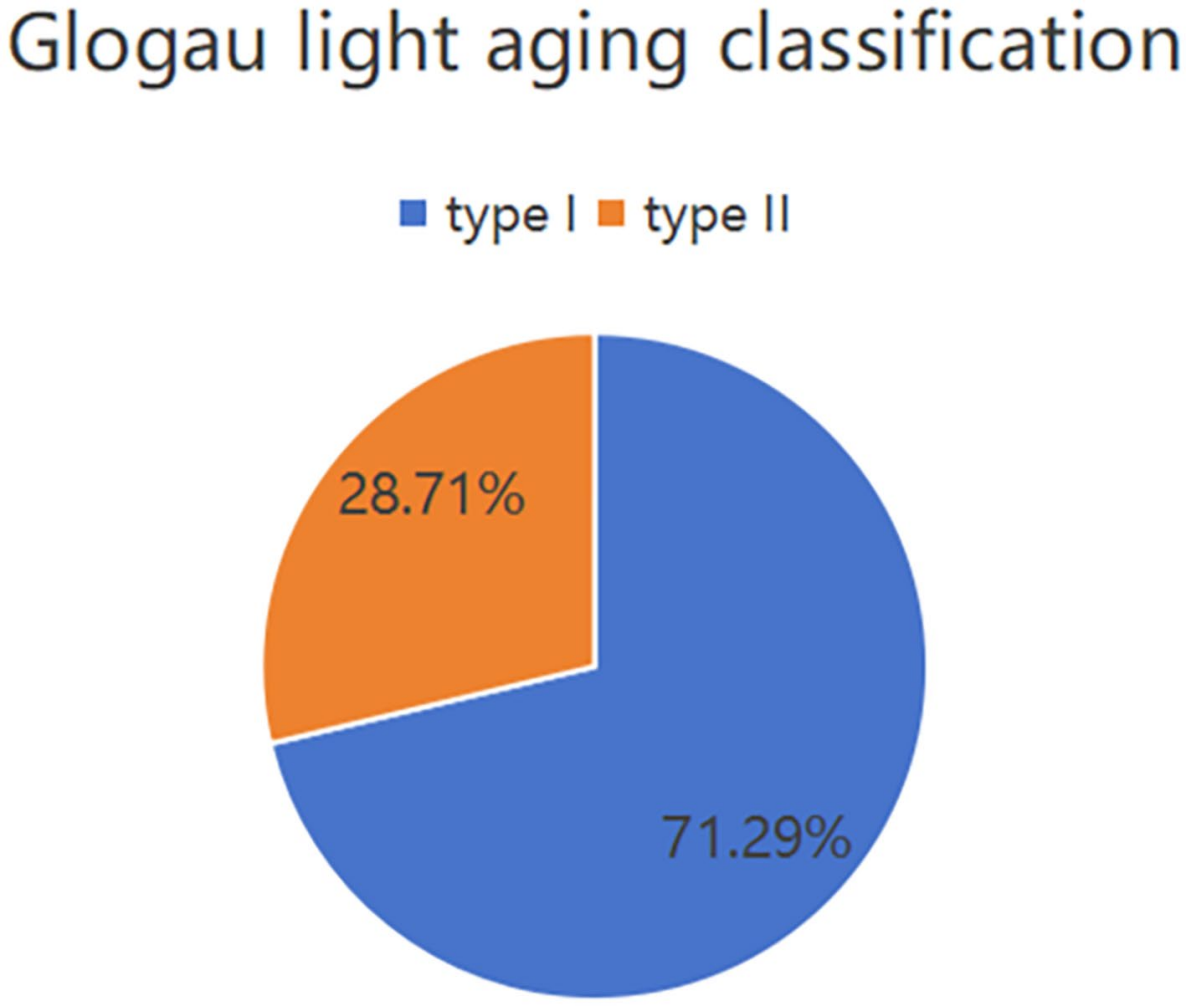
Distribution of patients included in the study according to the Glogau photoaging classification.

**Figure 4:**
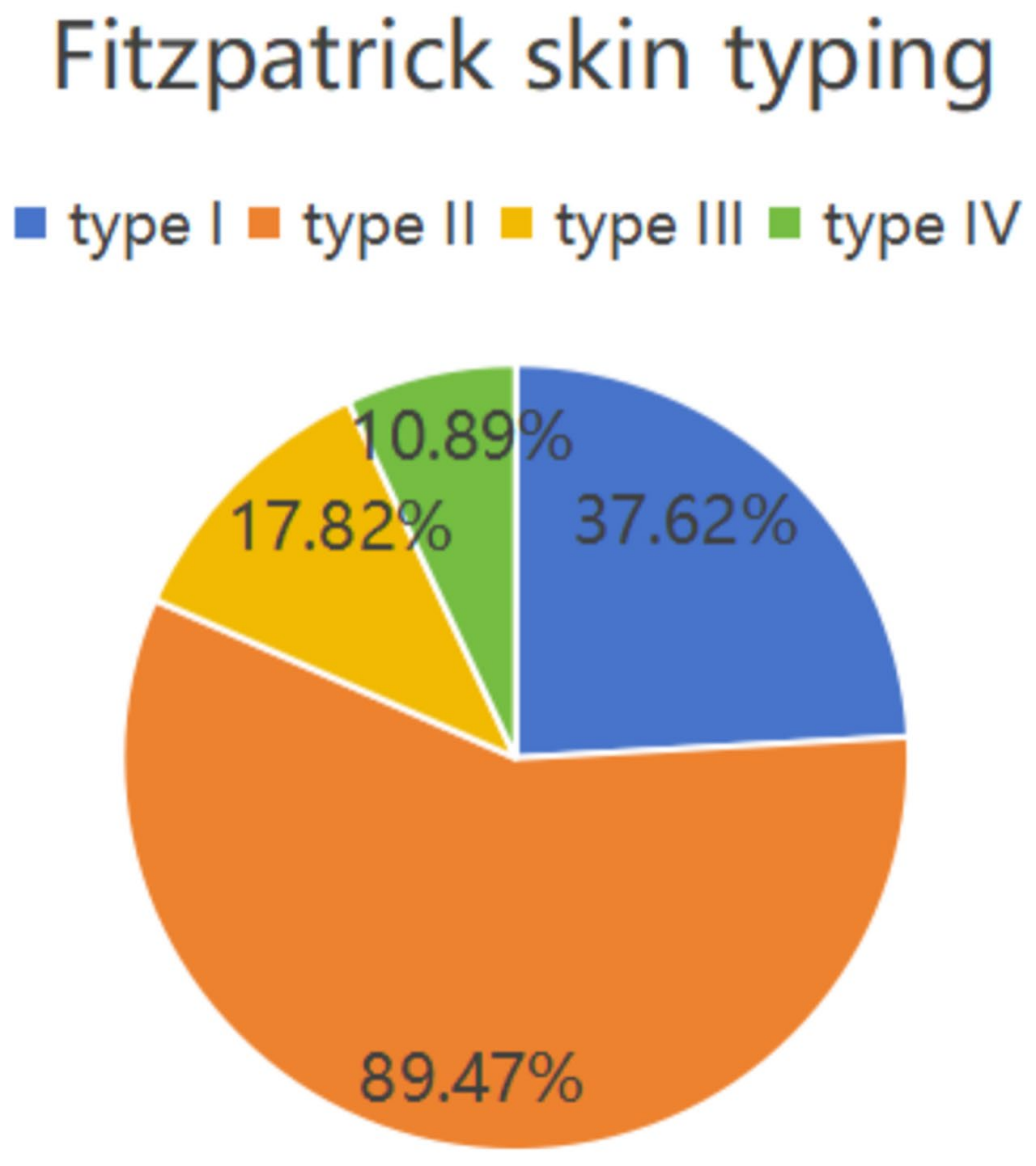
Distribution of patients included in the study according to the Fitzpatrick skin phototype.

Figure 3 displays the distribution of the Glogau photoaging classification among the 202 photodamaged young Asian female patients: 144 cases (71.29%) were classified as Grade I (mild photoaging), and 58 cases (28.71%) were classified as Grade II (moderate photoaging). No cases were classified as Grade III or IV. Figure 4 shows the distribution of Fitzpatrick skin phototypes: Type I comprised 76 cases (37.62%), Type II comprised 68 cases (33.66%), Type III comprised 36 cases (17.82%), and Type IV comprised 22 cases (10.89%). Notably, Types I and II collectively represented approximately 71% of the cohort, indicating predominant UV sensitivity in this young Asian female population. These findings suggest that skin photoaging in young Asian women predominantly manifests as mild-to-moderate in severity, with heightened UV sensitivity characteristic of lower Fitzpatrick skin phototypes.

### 3.3 Baseline comparability between the groups

Comparison of baseline characteristics between the groups revealed no statistically significant differences in age, place of residence, body mass index, education level, menarche age, daily sun exposure duration, Glogau photoaging classification, or Fitzpatrick skin phototype (all *p* > 0.05), confirming group comparability (Table 1).

**Table 1:** Baseline characteristic comparison between the groups.

Characteristics	RF-alone group (*n* = 80)	Combined group (*n* = 122)	t/χ^2^	*P*
Age (years)	28.05 ± 5.46	28.58 ± 5.59	0.665	0.507
Residence (urban)	50 (62.50%)	72 (59.02%)	0.245	0.621
BMI (kg/m^2^)	22.37 ± 1.55	22.04 ± 1.49	1.515	0.131
Education (≥high school)	60 (75.00%)	80 (65.57%)	2.018	0.155
Menarche age (years)	13.20 ± 1.14	13.40 ± 1.25	1.151	0.251
Daily sun exposure (h)	3.95 ± 0.56	4.06 ± 0.61	1.294	0.197
Glogau Grade I	60 (75.00%)	84 (68.85%)	0.892	0.345
Fitzpatrick types I–II	57 (71.25%)	87 (71.31%)	0.589	0.899

Data are presented as mean ± SD or n (%). RF, radiofrequency; BMI, body mass index.

### 3.4 Clinical efficacy comparison

The total effective rate of improvement, reflecting overall clinical response, was significantly higher in the combined group than in the RF-alone group (95.08% vs. 78.75%, χ^2^ = 12.773, *p* < 0.001). The absolute risk difference was 16.33% (95% CI: 7.24–25.42%), favoring combined treatment. Table 2 presents the detailed efficacy distribution, showing raw counts for each response category: in the RF-alone group, 20 patients (25.00%) achieved an excellent response, 24 (30.00%) achieved a good response, 19 (23.75%) achieved a fair response, and 17 (21.25%) achieved a poor response; in the combined group, 46 patients (37.70%) achieved an excellent response, 38 (31.18%) achieved a good response, 32 (26.23%) achieved a fair response, and only 6 (4.92%) achieved a poor response.

**Table 2:** Clinical efficacy comparison between the groups.

Outcomes	RF-alone group (*n* = 80)	Combined group (*n* = 122)	χ^2^	*P*
Excellent (>75%)	20 (25.00%)	46 (37.70%)		
Good (51–75%)	24 (30.00%)	38 (31.18%)		
Fair (26–50%)	19 (23.75%)	32 (26.23%)		
Poor (≤25%)	17 (21.25%)	6 (4.92%)		
Total effective rate	63 (78.75%)	116 (95.08%)	12.773	<0.001

Total effective rate = (excellent + good + fair)/total × 100%. Risk difference: 16.33% (95% CI: 7.24–25.42%).

### 3.5 Symptom score comparison

**Figure 5:**
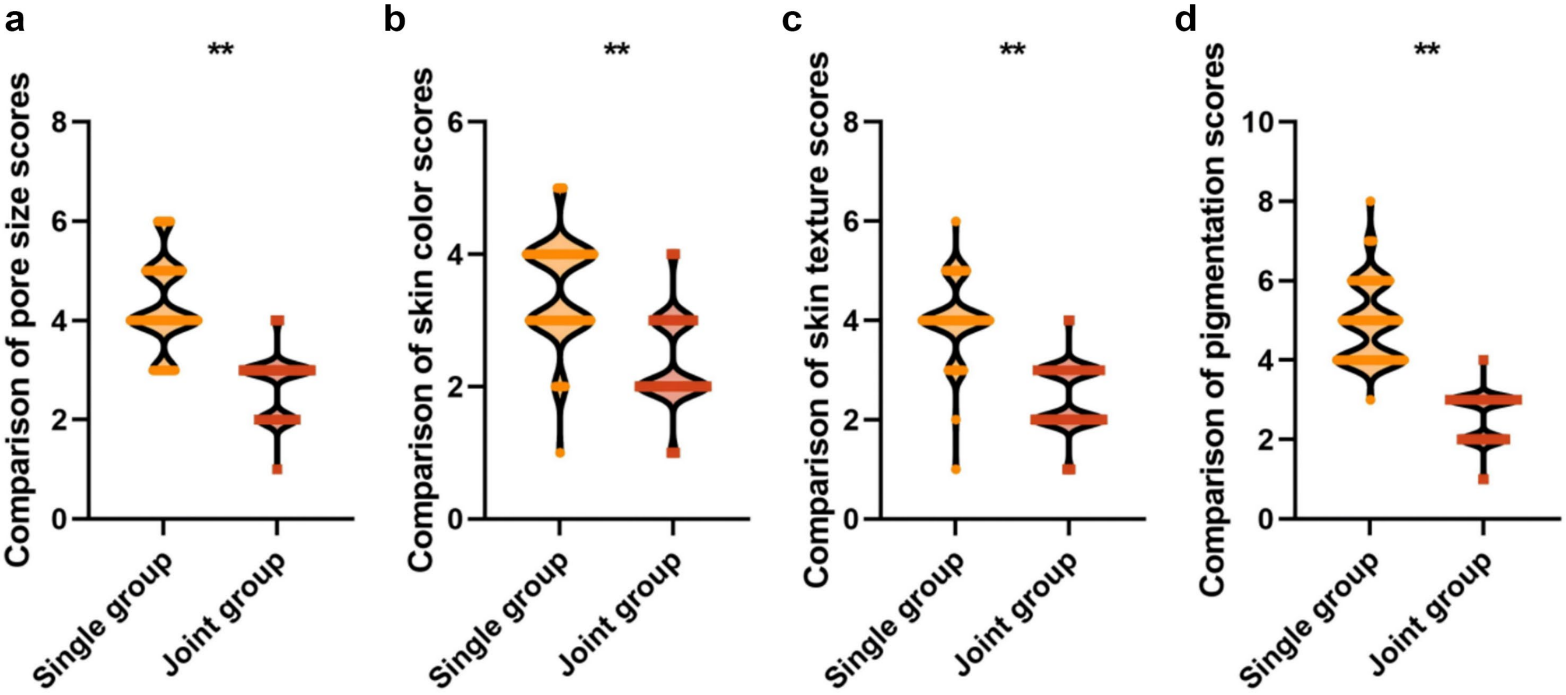
Comparison of post-treatment symptom scores between the groups. *p < 0.05 vs. RF-alone group. **Indicates *p* < 0.01.

Pre-treatment symptom scores showed no significant differences between the groups for pore size (6.20 ± 1.22 vs. 6.36 ± 1.35, *t* = 0.855, *p* = 0.393), hyperpigmentation (5.77 ± 1.08 vs. 5.63 ± 1.15, *t* = 0.867, *p* = 0.387), skin texture (5.48 ± 1.23 vs. 5.50 ± 1.25, *t* = 0.112, *p* = 0.911), or skin color (4.96 ± 1.20 vs. 4.87 ± 1.22, *t* = 0.516, *p* = 0.606) (Table 3). Following treatment, all symptom scores were significantly lower in the combined group: pore size (2.68 ± 0.56 vs. 4.30 ± 0.87, mean difference: −1.62, 95% CI: −1.85 to −1.39, *p* < 0.001), hyperpigmentation (2.59 ± 0.50 vs. 4.92 ± 0.97, mean difference: −2.33, 95% CI: −2.55 to −2.11, *p* < 0.001), skin texture (2.46 ± 0.60 vs. 4.02 ± 0.76, mean difference: −1.56, 95% CI: −1.76 to −1.36, *p* < 0.001), and skin color (2.39 ± 0.64 vs. 3.45 ± 0.77, mean difference: −1.06, 95% CI: −1.27 to −0.85, *p* < 0.001) (Figure 5).

**Table 3:** Comparison of pre-treatment symptom scores (x¯ ± s, points) between the two groups of young female patients with photodamaged skin.

Index		Single group (*n* = 80)	Joint group (*n* = 122)	*t-*value	*P-*value
Pore size	Prior treatment	6.20 ± 1.22	6.36 ± 1.35	0.855	0.393
Pigmentation	Prior treatment	5.77 ± 1.08	5.63 ± 1.15	0.867	0.387
Skin texture	Prior treatment	5.48 ± 1.23	5.50 ± 1.25	0.112	0.911
Skin color	Prior treatment	4.96 ± 1.20	4.87 ± 1.22	0.516	0.606

### 3.6 GPS score comparison

**Figure 6:**
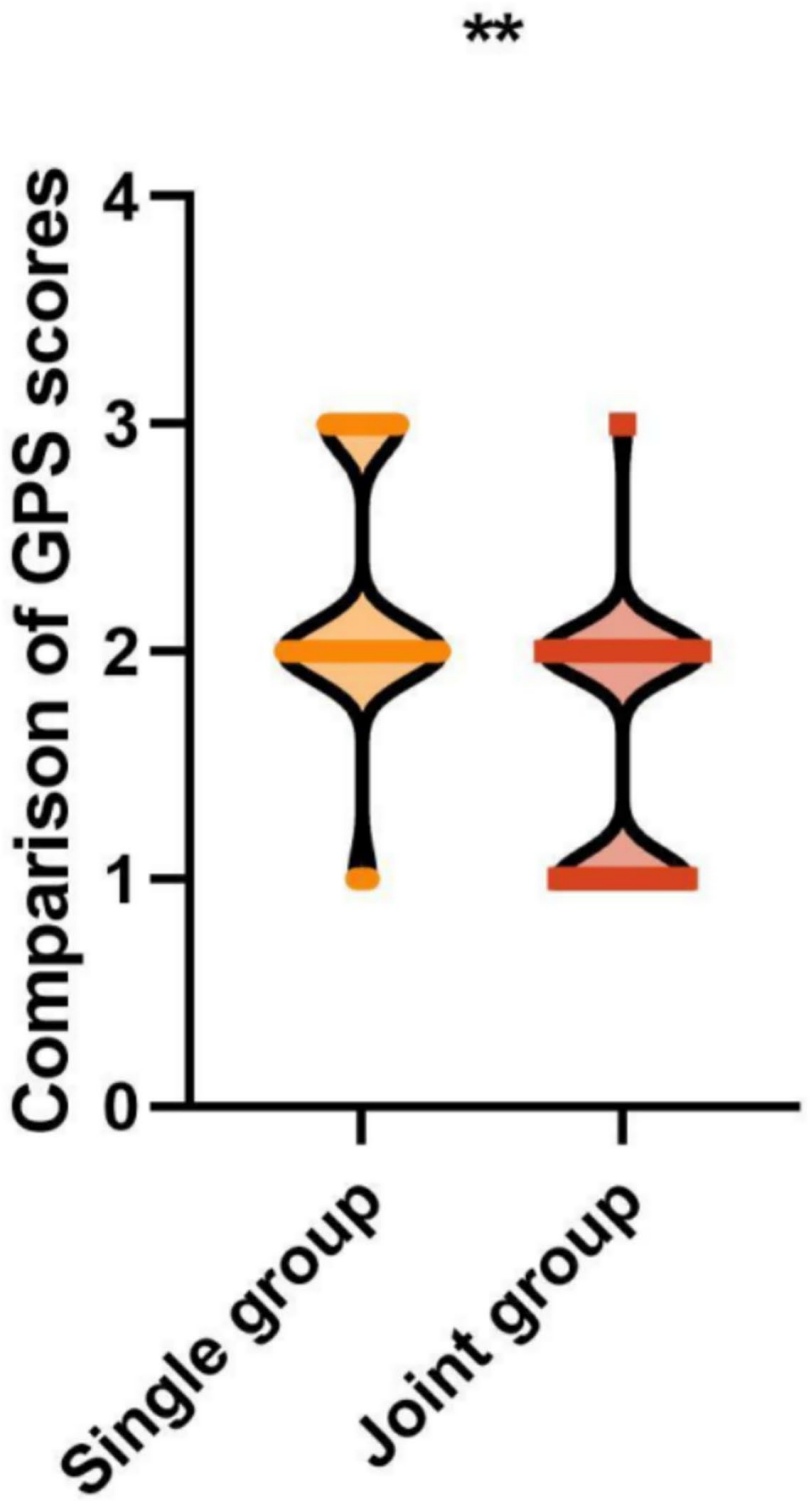
Comparison of GPS scores between the groups. GPS, global photoaging score; *p < 0.001 vs. RF-alone group. **Indicates *p* < 0.01.

Pre-treatment GPS scores were comparable between the groups (2.74 ± 0.56 vs. 2.80 ± 0.64, *t* = 0.688, *p* = 0.492). Post-treatment GPS scores were significantly lower in the combined group than the RF-alone group (1.58 ± 0.50 vs. 2.24 ± 0.56, *t* = 8.694, *p* < 0.001), with a mean difference of −0.66 (95% CI: −0.80 to −0.52), indicating superior global photoaging improvement with combined treatment (Figure 6).

### 3.7 Adverse reactions

The incidence of adverse reactions did not differ significantly between the groups (RF-alone group: 3.75% vs. combined group: 4.10%, χ^2^ = 0.016, *p* = 0.899; risk difference: 0.35, 95% CI: −4.38 to 5.08%) (Table 4). Observed adverse events included skin dryness, pruritus, burning sensation, erythema, and post-inflammatory hyperpigmentation, all of which resolved spontaneously within 1–2 weeks with conservative management. No serious adverse events requiring treatment discontinuation occurred in either group.

**Table 4:** Adverse reaction comparison between the groups.

Adverse events	RF-alone group (*n* = 80)	Combined group (*n* = 122)	χ^2^	*P*
Skin dryness	1 (1.25%)	1 (0.82%)		
Pruritus	1 (1.25%)	1 (0.82%)		
Burning sensation	0 (0.00%)	1 (0.82%)		
Erythema	0 (0.00%)	1 (0.82%)		
PIH	1 (1.25%)	1 (0.82%)		
Total	3 (3.75%)	5 (4.10%)	0.016	0.899

PIH, post-inflammatory hyperpigmentation. Risk difference: 0.35% (95% CI: −4.38 to 5.08%).

## 4 Discussion

Historically considered a concern primarily for middle-aged and elderly women, photoaging has increasingly affected younger populations due to environmental and behavioral changes. Salminen et al. (15) demonstrated that UV-induced inflammation and immunosuppression accelerate aging processes across all age groups. Krutmann et al. (16) emphasized that genetic predisposition, dietary patterns, lifestyle factors, and skincare habits collectively influence the photoaging trajectory, with inadequate photoprotection contributing to premature aging even in young women. Our findings substantiate this trend: among the 202 young Asian women (aged 18–35 years), photoaging predominantly manifested as Glogau Grades I–II (71.29 and 28.71%, respectively), with most patients exhibiting Fitzpatrick Types I–II (71.28% combined), indicating the heightened UV sensitivity characteristic of fairer Asian skin phenotypes.

The principal finding of this study is that non-ablative bipolar RF combined with ablative fractional CO₂ laser significantly outperformed RF monotherapy across multiple efficacy endpoints. The combined treatment achieved a total effective rate of 95.08% compared with 78.75% with RF alone (risk difference: 16.33, 95% CI: 7.24–25.42%, *p* < 0.001), representing a clinically meaningful improvement. Post-treatment symptom scores showed substantially greater reductions in the combined group for pore size (mean difference: −1.62), hyperpigmentation (−2.33), skin texture (−1.56), and skin color (−1.06), all with narrow confidence intervals, indicating precise effect estimation. Similarly, GPS scores showed significantly greater improvement in the combined group (mean difference: −0.66, 95% CI: −0.80 to −0.52).

These findings align with and extend previous research supporting multimodal photoaging treatment. Peters et al. (17) reported that the combination of 1,440-nm and 1927-nm non-ablative fractional lasers with monopolar RF provided superior outcomes for facial laxity, texture, and pigmentation compared with single-modality approaches. Lee et al. (18) demonstrated synergistic effects of fractional microneedling RF on senescence-induced skin hyperpigmentation. Long et al. (19) found that fractional laser combined with bipolar RF effectively treated atrophic facial acne scarring. Our results corroborate these findings while specifically demonstrating efficacy in young Asian women with early photoaging, a population previously underrepresented in studies of combination therapy.

The mechanistic rationale for combination superiority lies in the complementary therapeutic targets of each modality. Non-ablative bipolar RF generates volumetric dermal heating (40–42 °C), inducing immediate collagen contraction and subsequent neocollagenesis through fibroblast stimulation and heat-shock protein activation (6, 20). The bipolar electrode configuration ensures controlled energy deposition within the reticular dermis, addressing deep tissue laxity without epidermal disruption. In contrast, ablative fractional CO₂ laser creates controlled microthermal zones of tissue vaporization, triggering wound-healing cascades that remodel both collagen and elastin fibers while promoting epidermal regeneration (9, 21). The fractional approach spares intervening tissue, enabling rapid re-epithelialization and minimizing downtime. By combining these modalities in an alternating protocol (RF on weeks 1, 3, 5, 7, 9, 11, 13, and 15; CO₂ laser on weeks 2, 6, 10, and 14), we aimed to maximize collagen stimulation across different dermal depths while allowing adequate recovery between interventions.

The safety analysis revealed no significant difference in adverse reaction rates between the groups (4.10% vs. 3.75%, *p* = 0.899), with a risk difference of only 0.35% (95% CI: −4.38 to 5.08%). All adverse events were mild, self-limiting, and resolved within 1–2 weeks. This favorable safety profile is particularly relevant for Asian skin, which carries elevated PIH risk following laser and energy-based treatments (13). Our low PIH incidence (0.82% in the combined group) likely reflects the conservative energy parameters used, comprehensive photoprotection protocols, and the fractional delivery approach minimizing bulk thermal injury. Pour Mohammad et al. (22) similarly reported acceptable safety profiles when combining laser modalities with appropriate parameter settings in skin rejuvenation procedures.

Several practical considerations emerge from our findings. First, the Fitzpatrick phototype assessment should guide treatment planning in Asian populations; Types I–II patients, constituting the majority of our cohort, may tolerate more aggressive parameters than Types III–IV patients. Second, strict photoprotection (SPF 30 + sunscreen, physical barriers, and sun avoidance) throughout the treatment course is essential to minimize PIH risk and optimize outcomes. Third, antiviral prophylaxis should be considered for patients with a history of herpes simplex, as the fractional CO₂ laser treatment has the potential to trigger reactivation. However, this was an exclusion criterion in our study. Fourth, the alternating treatment schedule we used provides a practical framework for combination therapy, although the optimal sequencing and intervals warrant further investigation.

This study has several limitations warranting acknowledgment. First, the retrospective single-center design introduces potential selection bias and limits causal inference; patients self-selected the treatment modality based on preferences, cost considerations, and physician recommendations rather than randomization. Second, the 4-week post-treatment follow-up may not capture long-term efficacy or delayed adverse events; photoaging treatment effects typically evolve over months while collagen remodeling matures. Third, patient-reported outcome measures (PROs) assessing satisfaction, impact on quality of life, and perceived improvement were not systematically collected, limiting insights into subjective treatment benefits. Fourth, despite detailed RF and CO₂ laser parameter documentation, some procedural variations inherent to real-world clinical practice may introduce heterogeneity. Fifth, the absence of a fractional CO₂ laser monotherapy control group precludes direct comparison of individual modality contributions. Sixth, the sample size, while adequate for detecting observed effect sizes, may lack sufficient power for subgroup analyses (e.g., by Fitzpatrick type or Glogau grade). Finally, a cost-effectiveness analysis was not performed, which is relevant given that combination treatment involves additional procedure costs and patient time commitment.

Future prospective randomized controlled trials should address these limitations by incorporating: (1) randomized allocation to RF alone, fractional CO₂ laser alone, and combination treatment; (2) extended follow-up periods of at least 6–12 months; (3) validated PRO instruments; (4) standardized photography protocols enabling objective image analysis; (5) histological assessment in a subset of patients; (6) subgroup analyses stratified by baseline photoaging severity and skin phototype; and (7) health economic evaluation. A multicenter collaboration would enhance generalizability, particularly important given the geographic variation in Asian skin phenotypes and environmental UV exposure patterns.

## 5 Conclusion

This retrospective study characterizing photoaging in young Asian women demonstrates that early photoaging predominantly manifests at mild-to-moderate severity (Glogau Grades I–II) with heightened UV sensitivity (primarily Fitzpatrick Types I–II). Non-ablative bipolar radiofrequency combined with ablative fractional CO₂ laser provides significantly superior clinical efficacy compared to RF monotherapy, achieving a 16.33% absolute improvement in the total effective rate (95% CI: 7.24–25.42%) without increasing adverse events. For clinicians, this combination approach using conservative energy parameters (RF: 40.68 MHz, 50–120 W, target 40–42 °C; CO₂ laser: 20–28 mJ, 8–15 J/cm^2^, and 100–200 MTZs/cm^2^) in an alternating schedule offers an effective and safe treatment strategy for early photoaging management in young Asian women. Patients can expect an approximately 95% likelihood of meaningful improvement with minimal risk of complications when adhering to photoprotection guidelines. Prospective randomized trials are warranted to further optimize treatment protocols and establish long-term efficacy.

## Data Availability

The original contributions presented in the study are included in the article/supplementary material, further inquiries can be directed to the corresponding author.
